# Traumatic Injury and Complete Ligation of the Inferior Vena Cava: A Case Report

**DOI:** 10.7759/cureus.44977

**Published:** 2023-09-10

**Authors:** Hind Alhalyan, Abdulla Nidal, Fatma Almoosawi, Yousif Habib Al Abboudi

**Affiliations:** 1 Department of Trauma & Orthopaedics, Rashid Hospital, Dubai Academic Health Corporation, Dubai, ARE; 2 Department of Surgery, Rashid Hospital, Dubai Academic Health Corporation, Dubai, ARE; 3 Department of General Surgery, Rashid Hospital, Dubai Academic Health Corporation, Dubai, ARE

**Keywords:** damage control laparotomy, ligation, general surgery, trauma, inferior vena cava injury

## Abstract

This case report describes an occurrence of a traumatic injury to the inferior vena cava (IVC) secondary to penetrating trauma. A 37-year-old male patient presented to the emergency department (ED) after sustaining a stab wound to the mid back. The patient was transferred directly to the operating theatre (OT) and underwent an emergency exploratory laparotomy. A through-and-through IVC injury at the level of the entry to the liver bed was identified. Surgical repair was attempted but failed, followed by eventual IVC ligation. The patient was shifted to the intensive care unit postoperatively for damage control resuscitation while the abdomen was temporarily closed. He later required a re-look procedure for definitive treatment, after which he could mobilize without assistance and return home. This case highlights the different surgical approaches to an IVC injury and examines postoperative complications and their management.

## Introduction

Inferior vena cava (IVC) injuries are relatively rare, life-threatening injuries that result from trauma. Managing this type of injury is often challenging as it depends on the type of trauma, the extent of the damage to the vessel, the patient's condition, concomitant injuries, and the availability of the necessary expertise and postoperative intensive care unit (ICU) care [1]. The multifaceted nature of IVC injuries underscores the complexity of their management. Swift and effective interventions are paramount to prevent dire hemorrhagic outcomes; however, the approach must be meticulously tailored to the specific circumstances of each case. This case report discusses a case of IVC injury resulting from a penetrating injury to the back and the different modalities of surgical management.

## Case presentation

**Figure 1:**
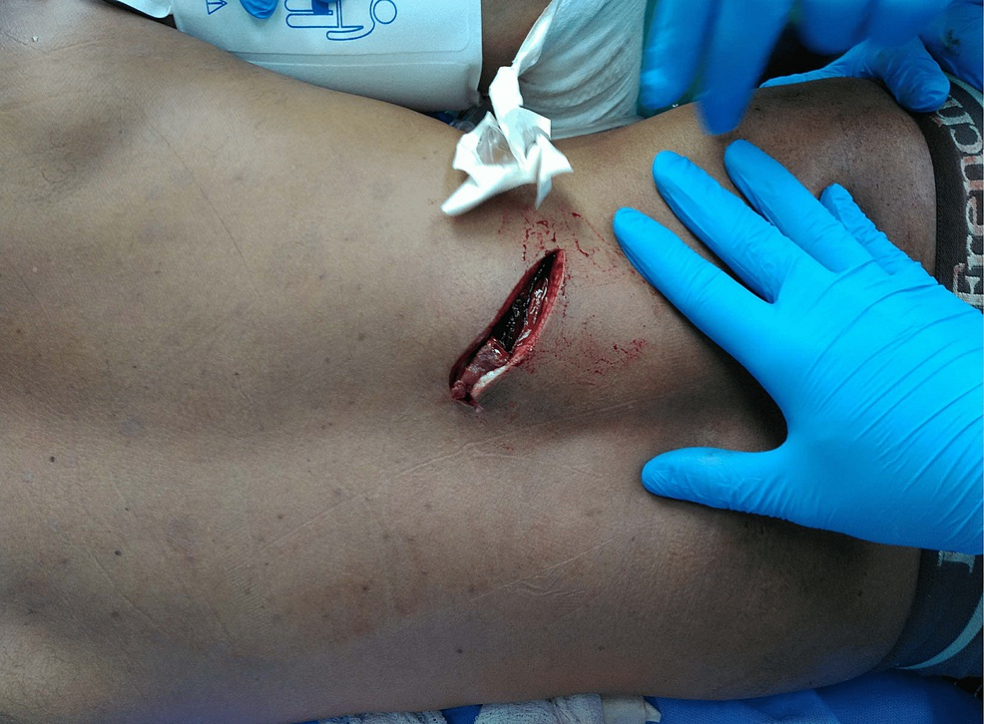
A 12 cm deep penetrating mid-back stab wound

A 37-year-old male not known to have any co-morbidities presented to the emergency department (ED) after sustaining a stab injury to the mid-back just under the tip of the right scapula (Figure 1) and multiple lacerations to both forearms. Initially, the patient had a Glasgow Coma Scale (GCS) of 14/15 and was conscious and oriented but confused, with airway maintained and bilateral equal air entry. He was normotensive but tachycardic. He received initial fluid resuscitation with lactated Ringer’s solution. On inspection, a 12 cm wound was seen in the mid-back just under the tip of the right scapula, as well as a superficial laceration of the right forearm and a deep 15cm laceration on the volar aspect of the left forearm, injuring the muscle and tendons beneath. Extended Focused Assessment with Sonography for Trauma (EFAST) scan was grossly positive, revealing fluid in Morrison’s pouch, the left lower quadrant, and the pelvic cavity but free chest cavity, normal pleural glide. The patient’s hemoglobin and lactate levels were 10.8 g/dL and 4.4 mmol/L on the arterial blood gas metabolic panel, respectively. He was planned to undergo an urgent computerized tomography (CT) scan when he suddenly developed hypotension of 60/30 mmHg with tachycardia of 130 bpm. Three units of uncross-matched type O negative packed red blood cells were readily available in the ED, and more fluids and tranexamic acid were given. He transiently responded to the resuscitation efforts before his blood pressure dropped again. He was deemed too unstable for a repeat primary survey or CT and was shifted directly to the OT.

The patient underwent an urgent exploratory midline laparotomy. A hemoperitoneum of 6 liters was encountered and evacuated, and all four quadrants were packed. The bleeding site was found to be the inferior surface of the liver. The ascending colon was mobilized medially, and the retroperitoneum was exposed via a Cattell-Braasch maneuver technique. The coronary ligament of the liver was transected, and a through-and-through injury to the IVC was identified, which was isolated, and a vessel loop was applied distally. Intraoperatively, the patient went into cardiac arrest. The intra-abdominal aorta was clamped at the suprahepatic area as it exited from the diaphragm, after which cardiopulmonary resuscitation (CPR) was started. Three cycles of CPR later, the return of spontaneous circulation was achieved.

The IVC was clamped, and the vascular surgery team joined the procedure. The IVC injury was further explored and dissected and was found to be beyond repair. The IVC was ligated, with two ligatures proximally and two distally. An abdominal drain was inserted, and the abdomen was temporarily closed.

Postoperatively, the patient was transferred to the ICU for damage control resuscitation. He remained intubated, tachycardic, and hypotensive. The patient then became anuric and developed an acute kidney injury (AKI), for which he received continuous renal replacement therapy. On subsequent examination, in the morning of the next day, there was decreased air entry on the right side and a tense abdomen. A chest X-ray revealed pleural effusion on the right side, with the mediastinum shifted to the contralateral side. A chest drain was inserted, which drained three liters of blood. The cardiothoracic surgery team was contacted and took the patient to OT for an urgent right-sided exploratory thoracotomy on the same day (one day post initial exploratory laparotomy). The posterior mediastinal pleura was found to be injured, and five liters of blood were drained from the thoracic cavity. As there were no signs of active bleeding, the remainder of the collection was evacuated, and the cavity was packed.

Two days after the initial exploratory laparotomy, the patient was taken for a re-look laparotomy. There were signs of active oozing of blood around the IVC injury site. A retroperitoneal hematoma was observed, which has not expanded since the previous surgery and has not been explored. 20 cm of small bowel wall was found to be gangrenous in patches 10 cm proximal to the ileocecal valve, which was resected but not anastomosed. A hemostatic agent (Floseal) was applied to the area of the IVC injury in an attempt to control the oozing blood. The surrounding area was packed, and another abdominal drain was inserted in the right upper quadrant. The abdomen was again temporarily closed and planned for another re-look. In the following days, the patient was taken for re-look laparotomy with general surgery cardiothoracic surgery. The gallbladder was found to be gangrenous. An open cholecystectomy was done, and the bowel looked healthy, so side-to-side anastomosis of the previously resected bowel was completed, and finally, definitive abdomen closure was performed. The cardiothoracic surgery team identified and suctioned a pericardial effusion, and the thoracic pack was removed. Lastly, once the patient was stable with an INR of 1.26, the orthopedic-trauma surgery team explored the wrist lacerations and dealt with the injuries accordingly (nerve and tendon injuries).

Approximately two weeks after the IVC ligation and index surgery, and while being on daily gastrointestinal prophylaxis (pantoprazole 40mg), the patient had an episode of massive hematemesis accompanied by melena and a serial complete blood count (CBC) revealed a significant drop in hemoglobin levels to 7.8 g/dL from a baseline of 10 g/dL. The patient received tranexamic acid and two blood units until he underwent an upper gastrointestinal endoscopy. The esophagus contained a high volume of blood, under which multiple ulcerations were seen at 28 cm and 35 cm distal to the incisor teeth, which were clipped.

**Figure 2:**
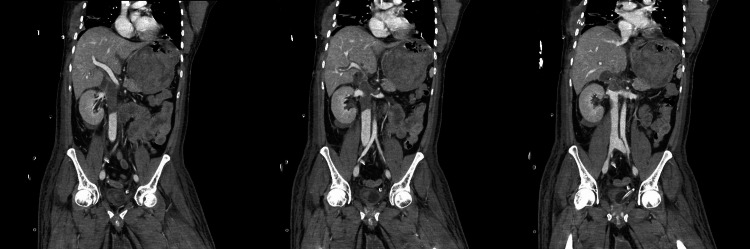
Multiple cuts of a CT abdomen showing a large thrombus of the distal IVC sparing the renal vessels and a blood-filled stomach. IVC: Inferior vena cava

The remainder of the patient’s hospital stay was unremarkable. He continued to receive ICU support until the extubation, after which he was shifted to the general ward. He gradually regained his kidney and liver functions. Before discharge, the patient underwent a CT of the chest and abdomen as part of his tertiary survey, which revealed a large thrombus of the distal IVC sparing the renal vessels (Figure 2).

Upon discharge, the patient was vitally stable with no active complaints. His liver function tests were normalizing (bilirubin: 0.9 mg/dL, alkaline phosphatase: 249 U/L down-trending from 325 U/L, ALT: 34 U/L, albumin: 3.9 g/dL). He was mobilizing with minimal support and tolerating oral feeding. The patient was advised to follow up with the general surgery clinic to assess his general condition, liver function tests, the need for anticoagulation, and plan appropriate physiotherapy and recuperation; however, the patient wished to continue his management in his home country.

## Discussion

Abdominal vascular injury (AVI) is a leading cause of non-compressible torso hemorrhage, accounting for up to 40% of all cases, as well as 8-25% of vascular trauma cases. IVC and iliac vein injuries account for the majority of AVIs involving veins [1]. IVC injuries have a reported mortality rate of 21-100%, depending on the site and extent of the injury [2]. This type of injury is seen with both blunt and sharp force trauma, where blunt force injuries are associated with higher mortality [2,3] and usually require more aggressive resuscitation and longer hospital stays [1]. IVC injuries are classified according to the anatomic level of the injury, as suprahepatic, infrahepatic, suprarenal, or infrarenal; however, there is no standardized classification. Suprarenal IVC injuries are associated with higher mortality when compared to injuries at the infrarenal levels, which carry the lowest mortality rates [1,3].

Patients are commonly hemodynamically unstable upon presentation, mainly when other concomitant injuries occur. In some cases, the bleeding is contained by a retroperitoneal tamponade, and patients are hemodynamically stable, as in our case. The FAST scan is used initially to evaluate for AVIs, while CT angiography is more accurate for visualizing AVIs [1].

Open surgery is the standard for AVIs in hemodynamically unstable patients with active hemorrhage evident on examination or imaging. Intra-abdominal retroperitoneal vascular structures are divided into three zones: Zone 1 contains the aorta, IVC, celiac trunk, and the superior and inferior mesenteries arteries; Zone 2 contains the renal vessels; and Zone 3 contains the iliac arteries and veins.

A midline laparotomy is performed, and the hemoperitoneum is evacuated with packing of the cavity. Once the bleeding is under control, a massive transfusion protocol should be activated, and then further exploration should be undertaken [1]. Suspected IVC injuries are best approached with right medial visceral rotation [1].

There are multiple management modalities for AVIs, both endovascular and surgical approaches, including endovascular stent repair, primary repair (venorrhaphy), interposition graft, and IVC ligation [3,4]. Primary repair of the damaged vessel wall is the preferred management modality; however, it is not always possible in view of the presence of other injuries or associated coagulopathy or hemodynamic instability. In such cases, emergent ligation may be necessary [5]. It is important to note that in hypovolaemic and hypotensive patients, complete occlusion of the infrarenal IVC will further worsen the hypotension due to decreased venous return [6]. In the case of complete hepatic vascular isolation, patients commonly suffer worsening shock or cardiac arrest as the venous return to the heart decreases [1]. Suprarenal IVC should not be ligated as that would interrupt renal drainage and push the patient into renal failure. Where possible, primary repair is preferred. In massive or smash injuries, temporary ligation in a damage control situation may be attempted [1]. In this case and in that reported by Votanopoulos et al. (2009), the patient survived the suprarenal IVC ligation. Suprarenal IVC ligation carries a high mortality rate, and survival is dependent on pre-existing adequate collateral systems between the IVC and the azygos-hemiazygos and the ascending lumbar venous systems [2].

Postoperatively, patients with managed AVIs are prone to developing complications such as pneumonia, pulmonary embolism, compartment syndrome, and deep venous thrombosis. Moreover, most patients develop varying degrees of lower limb edema which subsides in 7-10 days [7]. Patients with IVC injuries have a higher incidence rate of venous thromboembolism (VTE). Those who receive prophylactic full anticoagulation are less likely to develop VTE as compared to those on other VTE prophylactic regimens [8]. Those who develop compartment syndrome may require four-compartment lower extremity fasciotomies [1].

## Conclusions

This case report demonstrates the importance of the quick recognition of IVC injuries and immediate resuscitative and damage control efforts, including the activation of massive transfusion protocols and ICU admission for damage control resuscitation. The choice of management approaches depends on the location and severity of the injury, the patient's condition, the presence of other injuries, and the availability of a multidisciplinary team. As there is no standardized classification for IVC injuries, it is challenging to compile guidelines for the management of such cases. Overall, IVC injuries have a high mortality rate, whereas suprarenal IVC injuries account for the largest part of the fatalities. Primary repair is preferred when possible; if not, then ligation of the vessel is indicated as a life-saving measure. The treating physician must be aware of the many life-threatening complications of IVC ligation including VTE, where full anticoagulation can prevent these events, AKI, and compartment syndrome, necessitating fasciotomies, and be ready to manage them promptly.
